# Temporal trends in dietary creatine intake from 1999 to 2018: an ecological study with 89,161 participants

**DOI:** 10.1186/s12970-021-00453-1

**Published:** 2021-06-30

**Authors:** Darinka Korovljev, Nikola Todorovic, Valdemar Stajer, Sergej M. Ostojic

**Affiliations:** Applied Bioenergetics Lab, Faculty of Sport and PE, University of Novi Sad, Lovcenska 16, Novi Sad, 21000 Serbia

**Keywords:** Diet, Creatine, NHANES, Infants, Children

## Abstract

**Introduction:**

We described here the annual variations in mean dietary creatine intake from 1999 to 2018 in U.S. children and adults using National Health and Nutrition Examination Survey (NHANES) database.

**Methods:**

Dietary intake information from ten consecutive rounds of NHANES (from 1999 to 2000 to 2017–2018) was extracted for a total of 89,161 respondents aged 0–85 years. Individual values for total grams of creatine consumed per day were computed using the average amount of creatine (3.88 g/kg) across all creatine-containing food sources.

**Results:**

The average daily intake of creatine across the entire sample was 0.70 ± 0.78 g (95% confidence interval [CI], from 0.69 to 0.71) and 13.1 ± 16.5 mg/kg body weight (95% CI, from 13.0 to 13.2). A significant negative trend for dietary creatine intake was found in infants (*r* = − 0.019; *P* = 0.042), and children and adolescents (*r* = − 0.024; *P* < 0.001).

**Conclusions:**

Our findings suggest a variation in dietary creatine intake in the U.S. population during the past 20 years, with young persons tend to consume fewer grams of creatine per day from 1999 onwards. Long-running studies are highly warranted to assess possible health consequences of variable creatine intake in human nutrition.

## Introduction

Creatine (methyl-guanidinoacetic acid) is an essential contributor to cellular bioenergetics that is available from animal-based foods and synthesized endogenously in the human body. Based on functional needs, creatine can be classified as a conditionally essential nutrient for humans [1], and ~ 1.0 g of creatine per day ought to be obtained from an omnivorous diet to maintain its normal daily turnover [2]. A few recent studies analyzed dietary creatine intake across various age groups at the populational level [3–5], reporting a substantial number of children and adults who fail to consume enough creatine from food. Other than this, no population-wide studies have evaluated dietary creatine intake changes over time and perhaps recognize temporal trends in creatine malnutrition over the past decades. Here, we explored annual variations in mean dietary creatine intake from 1999 to 2018 in U.S. children and adults, using National Health and Nutrition Examination Survey (NHANES) database.

## Methods

Dietary intake information from ten consecutive rounds of NHANES (from 1999 to 2000 to 2017–2018) was extracted for a total of 89,161 respondents aged 0–85 years. The participants were categorized into three age-specific subsamples, infants (0–1.9 years; *n* = 6490), children and adolescents (2–17.9 years; *n* = 30,184), and adults (18.0 + years; *n* = 52,487). To calculate creatine intake per each NHANES round, we identified creatine-containing foods (e.g., meat, poultry, fish, and mixtures) using 8-digit food codes from the U.S. Department of Agriculture entries for individual foods. We subsequently recorded the gram weight of each food component containing creatine and calculated the net intake of those foods for each participant by merging all relevant food items on a daily basis. Individual values for total grams of creatine consumed per day were computed using the average amount of creatine (e.g., 3.88 g/kg for meat-based sources) across all creatine-containing food sources. Daily intake of creatine for each subsample was quantified in absolute (grams) and relative amount (mg per kg body weight), with the rationale for calculating the relative amount of creatine was based off of the research from Candow and colleagues [6], showing a relative dosing strategy of creatine is effective at improving bone and muscle biology. The mean intake across NHANES rounds compared with one-way ANOVA, with Mann-Kendell test was employed to test temporal trends in creatine intake across annual rounds. Approval to conduct NHANES was granted by the National Center for Health Statistics Research Ethics Review, with informed consent obtained from all participants.

## Results

**Fig. 1:**
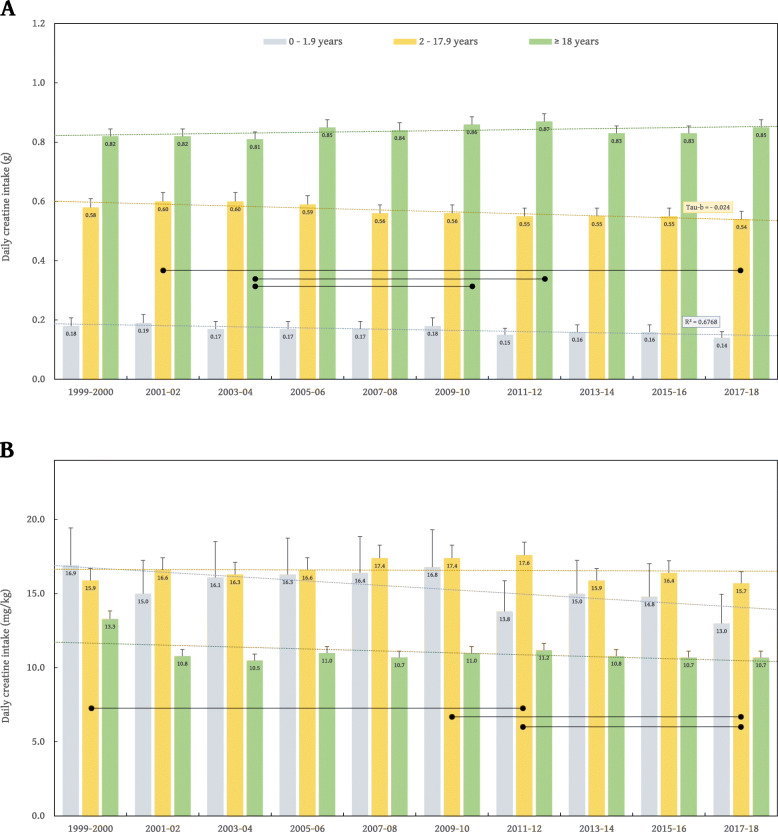
The mean dietary intake of creatine across ten NHANES rounds (1999–2000 to 2017–18) in three age-specific categories; the amounts are presented in grams (Panel **A**) and milligrams per kilogram of body weight (Panel **B**), with error bars indicate 95% confidence interval. Lines with circle ends indicate a significant difference between marked NHANES rounds at *P* < 0.05. Dashed lines are trendlines for each age category, with Kendall’s tau-b coefficient shown in case of significant trend detected (*P* < 0.05)

The average daily intake of creatine across the entire sample was 0.70 ± 0.78 g (95% confidence interval [CI], from 0.69 to 0.71) and 13.1 ± 16.5 mg/kg body weight (95% CI, from 13.0 to 13.2). The annual variation in creatine food intake is depicted in Fig. 1. One-way ANOVA revealed no significant differences between NHANES rounds in absolute and relative intakes of creatine for infants (*P* = 0.149 and *P* = 0.283, respectively). A significant difference between annual NHANES rounds was found for absolute creatine intake in children and adolescents (*P* < 0.001), with Tukey posthoc analysis shown significantly higher intake in NHANES 2001–02 as compared to NHANES 2017–18 (*P* = 0.028). A difference was also found for relative creatine intake in this subsample (*P* < 0.001), with posthoc analysis revealed significant differences between NHANES 1999–2000 and NHANES 2011–12 (*P* = 0.020), NHANES 2009–10 and NHANES 2017–18 (*P* = 0.045), and NHANES 2011–12 and NHANES 2017–18 (*P* = 0.020). The mean absolute intake of creatine across NHANES rounds was significantly different for adults (*P* = 0.008), with the intake was lower in NHANES 2003–04 as compared to both NHANES 2009–10 (*P* = 0.049) and NHANES 2011–12 (*P* = 0.021); no difference was found between NHANES rounds for the relative intake of creatine in adults (*P* = 0.074). A trend test shown a significant negative trend for absolute creatine intake in infants (*r* = − 0.019; *P* = 0.042) and children and adolescents (*r* = − 0.024; *P* < 0.001).

## Discussion

This is the first study describing temporal trends in dietary creatine intake at the populational level to the authors’ knowledge. We found that creatine consumption fluctuates among U.S. children and adults during the past two decades, with the absolute intake tended to decline in infants, children, and adolescents while the relative amount of creatine consumed kept relatively steady during this monitoring period. The highest intake of creatine among adults was reported during the NHANES 2011–12 round (0.87 g/day), yet the amount remains below recommended levels of 1.0 g/day for an average adult [2]. Besides, it appears that 68.6% of adults (35,983 out of 52,487 individuals) consumed less than one gram of creatine daily, indicating a relatively high proportion of inadequate dietary creatine intake in this age group. Such a prediction for non-adults remains unattainable since no dietary creatine requirements or recommendations for younger age are available at the moment.

During the 20-year period, dietary creatine intake decreased in U.S. infants, children, and adolescents. Our data also suggest that for each additional round of NHANES completed from 1999 to 2000 onwards, the expected amount of creatine consumed significantly decreased for 2.08 mg in infants, and for 3.22 mg in children and adolescents. Although this amount appears small, the trend might be of high clinical relevance owing to the fundamental role that dietary creatine plays in normal growth and health, with lower creatine availability appears to jeopardize young brain development in preclinical and clinical nutrition [7]. Whether dietary creatine shortfall affects children’s well-being currently remains unaddressed at the community-wide level. The possible factor contributing to the reduction of dietary creatine intake in youth might include lower meat consumption across U.S. households [8]. Since lean red meat, fish, and poultry are the primary dietary sources of creatine, eating less meat is likely accompanied by reduced dietary creatine exposure. This perhaps justifies backing diets rich in creatine-containing foods and low-dose supplementation or food fortification with creatine to optimize its dietary load in the general public [4]. Adding creatine to a regular diet normalizes creatine utilization and brain function in children with inborn errors of creatine metabolism [9]. Nevertheless, prospective studies must establish adequate dietary allowances for creatine to meet healthy persons’ needs across various life-stage groups.

The limitations of our study include the following: (*a*) the use of 24-h recall method to measure food consumption of NHANES participants; (*b*) implementing the identical amount of creatine across all creatine-containing meat-based foods while the creatine content can vary greatly across animal protein subgroups [3]; (*c*) not including non-meat creatine foods (e.g., milk and milk products) and nutritional supplements in computing daily creatine intake although their contribution to daily creatine intake appears relatively negligible [3]; (*d*) an inability to include pre-1999 NHANES data for more comprehensive comparisons, and (*e*) not accounting for endogenous creatine synthesis that contributes to total creatine turnover. Still, the mean daily intake of creatine in the current study appears similar to other reports using the NHANES database published recently [3–5]; a minor variation between studies may reflect different techniques employed to handle fragmented dietary entries and missing data, and non-identical sample composition and scaling. A few studies to date have combined or compared creatine intake data from several NHANES rounds across multiple years by maximizing sample size, which is the main advantage of current trial.

## Conclusion

In conclusion, our findings suggest a variation in dietary creatine intake among U.S. children and adults during the past 20 years, with young persons tend to consume fewer grams of creatine per day from 1999 onwards. Creatine consumption should be regularly monitored and quantified in individuals of all ages; future long-running studies are highly warranted to assess possible health consequences of variable creatine intake in human nutrition.

## Data Availability

Data described in the manuscript will be made available upon request.

## Abbreviations

NHANES: National Health and Nutrition Examination Survey
